# Interfering with the interaction between ErbB1, nucleolin and Ras as a potential treatment for glioblastoma

**DOI:** 10.18632/oncotarget.2343

**Published:** 2014-08-12

**Authors:** Yona Goldshmit, Sari Schokoroy Trangle, Yoel Kloog, Ronit Pinkas-Kramarski

**Affiliations:** ^1^ Department of Neurobiology, Tel-Aviv University, Ramat-Aviv, Israel; ^2^ Australian Regenerative Medicine Institute, Monash University, Australia

**Keywords:** nucleolin, Ras, ErbB1, FTS, AS1411, signal transduction

## Abstract

The three oncogenes, ErbB receptors, Ras proteins and nucleolin may contribute to malignant transformation. Previously, we demonstrated that nucleolin could bind both Ras protein and ErbB receptors. We also showed that the crosstalk between the three proteins facilitates anchorage independent growth and tumor growth in nude mice, and that inhibition of this interaction in prostate and colon cancer cells reduces tumorigenicity. In the present study, we show that treatment with Ras and nucleolin inhibitors reduces the oncogenic effect induced by ErbB1 receptor in U87-MG cells. This combined treatment enhances cell death, reduces cell proliferation and cell migration. Moreover, we demonstrate a pivotal role of nucleolin in ErbB1 activation by its ligand. Nucleolin inhibitor prevents EGF-induced receptor activation and its downstream signaling followed by reduced proliferation. Furthermore, inhibition of Ras by Salirasib (FTS), mainly reduces cell viability and motility. The combined treatment, which targets both Ras and nucleolin, additively reduces tumorigenicity both *in vitro* and *in vivo*. These results suggest that targeting both nucleolin and Ras may represent an additional opportunity for inhibiting cancers, including glioblastoma, that are driven by these oncogenes.

## INTRODUCTION

Glioblastoma is a highly aggressive class of tumors with hallmark features that include proliferation, genetic instability and chemoresistance [1]. Thus, the treatment of glioblastoma remains a challenging question. Several studies have identified epidermal growth factor receptor (EGFR also known as ErbB1/HER1) and its downstream signaling molecules as unregulated in glioblastoma [2]. Amplification of ErbB1 and alteration of its activity are important contributors to glioblastoma development [2]. Therefore, identification of proteins that regulate ErbB1 activation, degradation or part of its downstream signaling, may promote the development of new targeted therapy. Among the downstream effectors of ErbB1 is the Ras signaling pathway. The Ras family of small GTPases can transmit extracellular signals, which are initiated by the ErbB1, to regulate various cellular processes including cell proliferation, differentiation, motility and death [3]. Signals transmitted by activated Ras induce activation of multiple pathways [3-5]. Although Ras mutations are rare in glioblastoma [6], Ras can be activated by ErbB1, which is often over expressed in these tumors. Therefore, targeting Ras activation can potentially reduce malignancy. S-*trans, trans-*farnesylthiosalicylic acid (FTS; also known as Salirasib) is a synthetic Ras inhibitor that structurally resembles the carboxy-terminal farnesylcysteine group common to all Ras proteins. FTS acts as a Ras antagonist in cells and thereby reduces cellular Ras content [7].

Nucleolin is a ubiquitously expressed acidic phosphoprotein with key functions in transcription, synthesis and maturation of ribosomes [8, 9]. Nucleolin is involved in regulation of cell proliferation and cell growth [10, 11]. It is localized primarily in the nucleoli, but it is also found in the cytoplasm and on the cell surface of some types of cells [9, 12-14]. Nucleolin over expression is related with increased cell proliferation. Cell surface nucleolin is found in a wide range of tumor cells, and it is used as a marker for cancer diagnosis [15, 16]. Increased levels of cytoplasmic and cell-surface nucleolin have been demonstrated to correlate with malignancy grade and proliferation rate in glioblastoma [15]. Inhibition of cell-surface nucleolin and nucleolin activities, suppresses the growth of various cancer cells that may also express high levels/or activated Ras protein [8, 17-19]. In our previous studies, we identified non-nucleolar nucleolin as an ErbB receptor-interacting protein [20-23]. This interaction leads to ErbB1/EGFR receptor activation as well as to colony growth in soft agar [20]. In addition, recently we identified a crosstalk between nucleolin, ErbB1 and Ras proteins [22]. More recently, we have demonstrated that treatment of colon and prostate cancer cells with FTS and GroA (AS1411) inhibited cell growth and anchorage independent growth [24]. In the present study, we used U87-MG glioblastoma cells to examine the impact of Ras and nucleolin inhibition on ErbB1 and on the crosstalk between these three oncogenes. We demonstrated a pivotal role of nucleolin in activation of ErbB1 with or without its ligand EGF, in glioma cells. We showed that GroA (nucleolin inhibitor) reduced ErbB1/nucleolin interaction, affected EGF-induced ErbB1 activation and facilitated the degradation of ErbB1. In addition, GroA treatment significantly inhibited cell proliferation whereas FTS treatment increased cell death. Furthermore, the combined treatment of GroA and FTS inhibited cell growth, induced cell death and reduced cell motility. Moreover, the same treatments exhibited similar effects *in vivo*, namely, the combined treatment reduced ErbB1 phosphorylation and ErbB1/nucleolin interactions, induced cell death and reduced tumor volume in nude mice.

## RESULTS

### The effect of FTS and GroA on ErbB1 phosphorylation and ErbB1/nucleolin interaction

**Figure 1:**
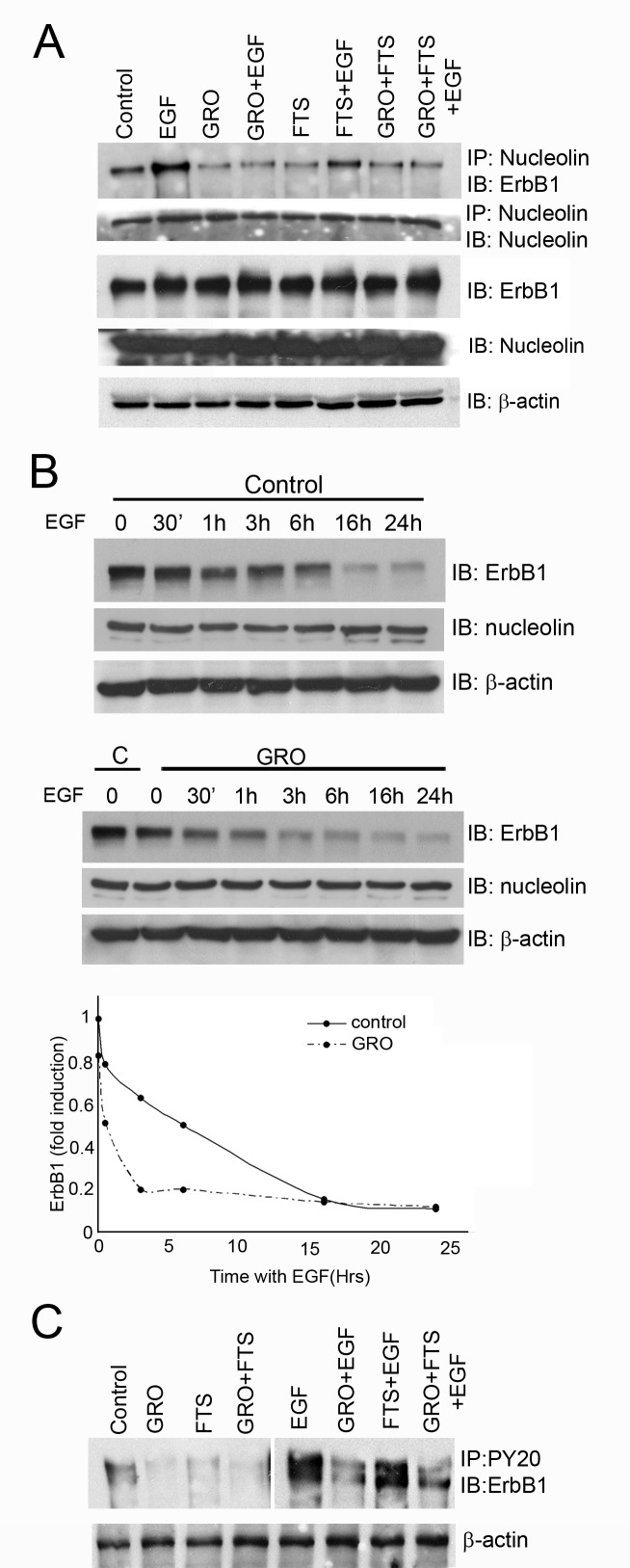
Ras and nucleolin inhibition reduces receptor nucleolin interaction and EGF induced ErbB1 phosphorylation A. U87-MG cells were treated with FTS 75μM, GroA 10μM or both for 48 hr, followed by EGF (100ng/ml) treatment for 30 min. Cell lysates were immunoprecipitated with anti nucleolin Ab and blotted with anti ErbB1 or with anti nucleolin Abs. As a control, total cell lysates were immunoblotted with anti ErbB1, anti nucleolin or anti-actin Abs. B. Cells were treated with GroA 10μM for 48 hr, followed by EGF (100ng/ml) treatments for the indicated time periods. Cell lysates were immunoblotted with anti-ErbB1, anti nucleolin and anti-actin Abs. Densitometric analysis of ErbB1 levels of representative experiment is presented as fold induction compared to the control at time=0. The experiment was repeated three times with similar results. C. lysates were precipitated with anti-PY20 and blotted with anti-ErbB1 antibodies. As a control, total cell lysates were immunoblotted with anti-actin Abs.

U87-MG cells express high levels of ErbB1 receptor and cell surface nucleolin [2]. Previously we have demonstrated a crosstalk between Ras, nucleolin and ErbB1 [21-24]. In order to test the effect of nucleolin and Ras inhibition on ErbB1 phosphorylation in glioblastoma cells, we used two inhibitors: FTS (salirasib) [25-27], a powerful Ras inhibitor, and GroA (AS1411), an aptamer that targets cell surface nucleolin [8, 28-30]. Cells were treated or untreated with FTS or GroA for 48 hr and then treated with or without EGF for 30 min. Cell lysates were subjected to immunoprecipitation with anti-nucleolin antibodies and blotted with anti ErbB1 antibodies. As shown, treatment with each drug alone or in combination reduced the interaction between the two proteins (Figure 1A). Furthermore, EGF increased the interaction between ErbB1 and nucleolin, whereas, GroA treatment alone or in combination with FTS treatment, inhibited EGF-induced elevation of ErbB1 and nucleolin interaction. Since GroA treatment affected ErbB1/nucleolin interaction with or without EGF, we next examined the time course of EGF-induced receptor degradation. As shown in Figure 1B, receptor degradation was enhanced by GroA treatment. Furthermore, as shown in Figure 1C, GroA treatment with or without FTS inhibited EGF-induced ErbB1 phosphorylation, which suggests that the interaction between the two proteins has a functional role. Thus, inhibition of cell surface nucleolin may affect its interaction with ErbB1 as well as the ligand-induced ErbB1 degradation or phosphorylation.

### FTS and GroA effect on MAPK and PKB/AKT signaling pathways

**Figure 2:**
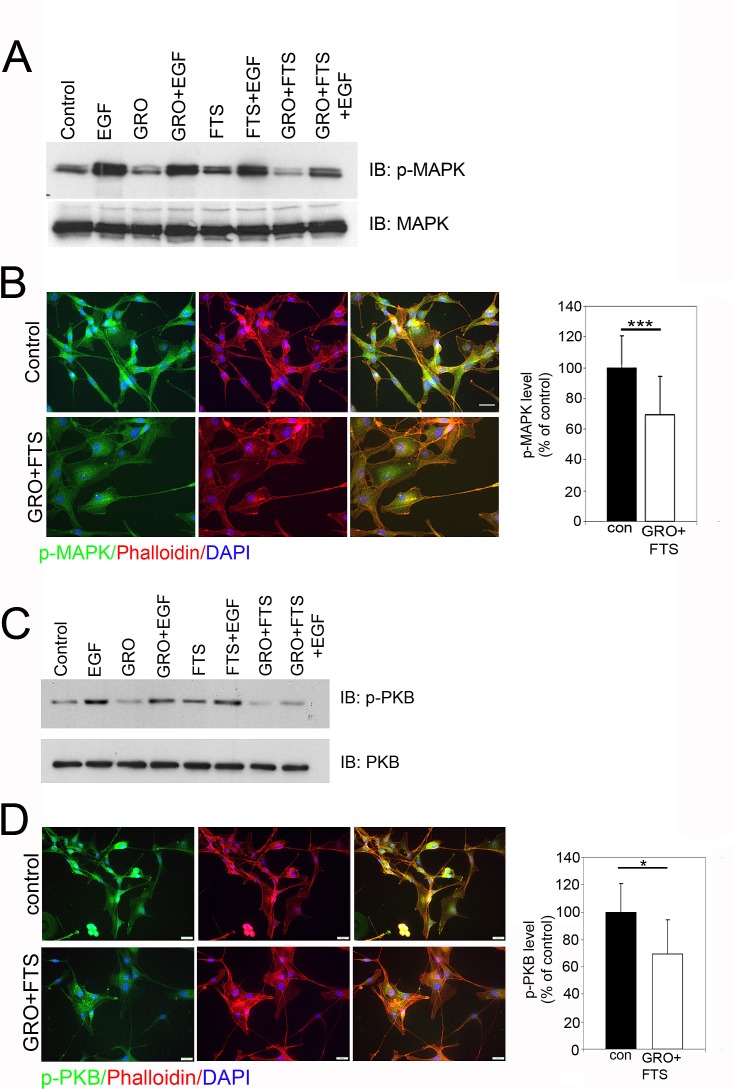
Ras and nucleolin inhibition reduces p-MAPK and p-PKB levels U87-MG cells were treated with FTS 75μM, GroA 10μM or both for 48 hr, followed by EGF (100ng/ml) treatment for 30 min (A and C). A. Total cell lysates were analyzed by western blot using anti p-MAPK. As a control, total cell lysates were immunoblotted with anti- MAPK. B. Cells were treated with FTS 75μM and GroA 10μM following staining with anti- pMAPK and actin (using phalloidin-rhodamine). Fluorescence levels were quantified by image J and were compared to the control (100%). C. Total cell lysates were analyzed by western blot using anti p-PKB. As a control, total cell lysates were immunoblotted with anti-PKB Abs. D. Cells were treated with FTS 75μM and GroA 10μM following by staining with p-PKB and actin staining using phalloidin-rhodamine. Fluorescence levels were quantified by image J and were compared to the control (100%). Results are mean ± SEM (n =3 experiments; ***p<0.001, two-tailed t-test, 95% confidence). Scale bars in B and D are 20μm.

Since inhibition of nucleolin and Ras affected receptor phosphorylation and receptor degradation, we next examined the combined effect of the drugs on ErbB1 downstream signaling (Figure 2). Two main pathways activated by ErbB1 and Ras are the mitogen activated protein kinase (MAPK) and protein kinase B (Akt/PKB). Combination of both drugs significantly inhibited both downstream pathways as detected either by Western blot or by immunostaining (Figure 2). Cells were treated with and without GroA in the presence or in the absence of FTS for 48 hr and then treated with EGF for 30 min. As shown, inhibition of Ras and nucleolin together reduced EGF-induced MAPK (Figure 2A) and PKB (Figure 2C) activation more effectively compared to each of the treatments alone. As demonstrated by immunostaining using anti-phosphorylated MAPK (Figure 1B) and PKB (Figure 1D), the activation of these pathways was significantly reduced. Moreover, co-staining with phalloidin, which stains actin filaments, demonstrated that the combined treatment affected cell morphology. Treated cells became more flattened and contained stress fibers (Figure 2B and D). Hence, the combined treatment can effectively inhibit Ras downstream signaling pathways, which may affect cell proliferation and viability.

### The effect of FTS and GroA on cell proliferation

**Figure 3:**
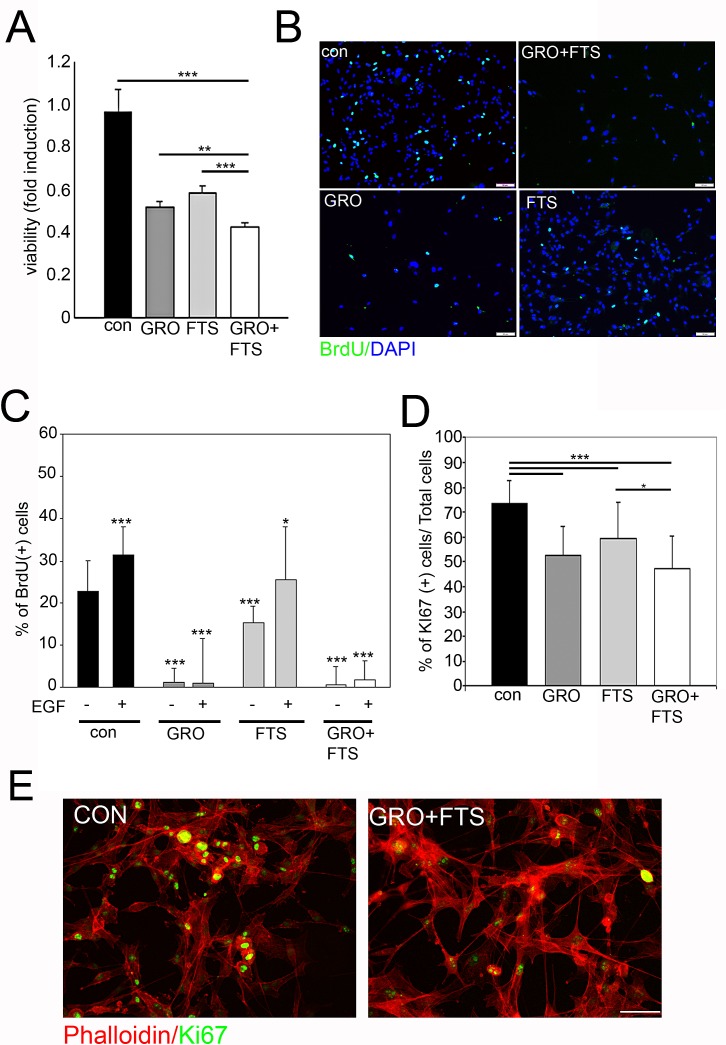
Ras and nucleolin inhibition reduces cell proliferation U87-MG cells were treated with FTS 75μM, in the presence or in the absence of 10μM GroA. A. Viability was tested after 5 days of treatment, using the methylene blue staining assay. Cell proliferation was assayed after 3 days of treatment by BrdU incorporation (B and C) and Ki67 staining (D and E). In each field the percentage of proliferating cells was estimated by counting the BrdU/Ki67-positive cells relative to the total number of cells (DAPI counts). Results are mean ± SEM (n =3 experiments; *p<0.05, **p<0.01, ***p<0.001, one-way ANOVA; 20-30 fields were analyzed per treatment) in A, C, D. Scale bars in B and E is 10μm.

In order to test the effect of nucleolin and Ras inhibition on cell growth and viability we first examined the ability of FTS and GroA to reduce the number of cells as determined by the methylene blue assay (Figure 3A). As shown, FTS and GroA each reduced the number of live cells, while the combined treatment was significantly more effective than each of the treatments alone. Since the number of cells was reduced following treatment, we further asked whether the drugs influence cell proliferation or cell death. Therefore, we examined cell proliferation using the Bromodeoxyuridine (BrdU) incorporation assay (Figure 3B and C). As shown, each treatment alone inhibited BrdU incorporation, but the GroA, as well as the combined treatments, were significantly more effective compared to FTS treatment alone (Figure 3C). Moreover, proliferation mediated by EGF was almost completely blocked by GroA treatment. This highlights the role of nucleolin as a regulator of cell proliferation via EGF-dependent ErbB1 activation. Similar results were also obtained by using the proliferation marker Ki67, which labels cells during the different phases of the cell cycle (Figure 3D and 3E). The combined treatment was significantly more effective in inhibition of cell growth compared to FTS treatment alone. However, there was no significant difference between the combined treatment and GroA treatment alone. This may suggest that GroA has a strong inhibitory effect on cell proliferation.

### The effect of FTS and GroA on cell death

**Figure 4:**
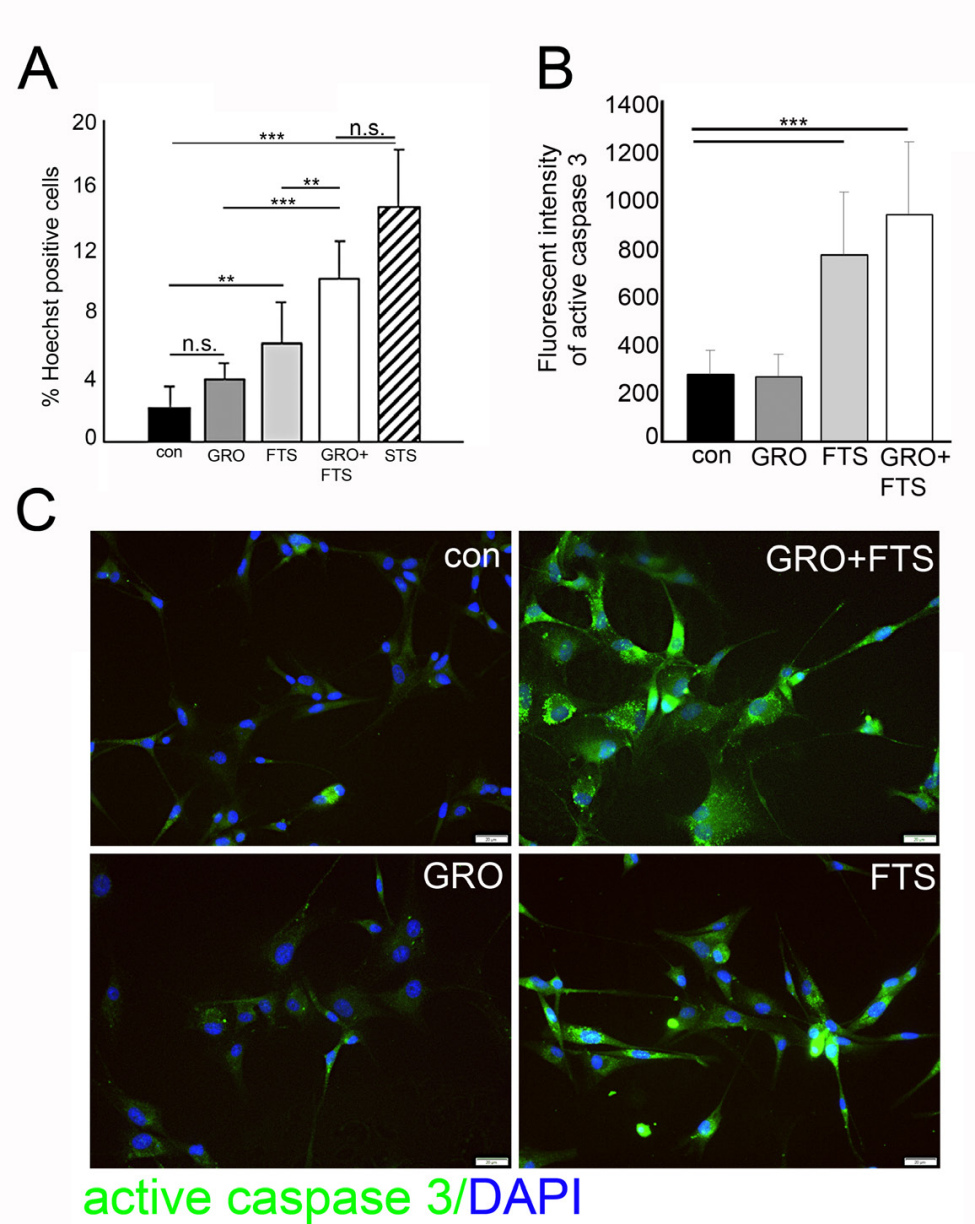
Ras inhibition increases cell death U87-MG cells were treated with FTS 75μM, in the presence or in the absence of 10μM GroA, for 3 days, in order to determine the number of dead cells. A. The treated cells were stained with the fluorescent DNA dye bisbenzimide (Hoechst). As a positive control, the cells were treated with 200 nM STS (staurosphorine). The percentage of dying cells was estimated by counting the Hoechst-positive cells relative to the total number of cells. B. Treated cells were stained with active caspase 3 and the intensity of the fluorescence was measured by image J software (20-30 fields were analyzed in each treatment). C. Representative images of the active caspase 3 staining. Results are mean ± SEM (n =3 experiments; n.s.= non significance, *p<0.05, **p<0.01, ***p<0.001, one-way ANOVA) in A and B. Scale bars in C is 20μm.

As described above, FTS and GroA co-treatment reduces cell number, which may result from cell growth inhibition but also from enhanced cell death. To determine the degree of cell death induced by the combined treatment we used the Hoechst dye exclusion assay, 3 days following treatments. As shown, we observed enhanced cell death following FTS treatment which was further increased when combined with GroA treatment as indicated (Figure 4A). As a positive control for cell death induction, we used staurosporine, a known apoptotic inducer. To further investigate cell death, we examined whether it is caspase dependent (Figure 4B and C). As shown, active caspase 3 levels were significantly elevated following FTS treatment and following the combined treatment. These results suggest that most of the cell death observed in the combined treatment is mediated by FTS.

### The effect of FTS and GroA on cell migration

**Figure 5:**
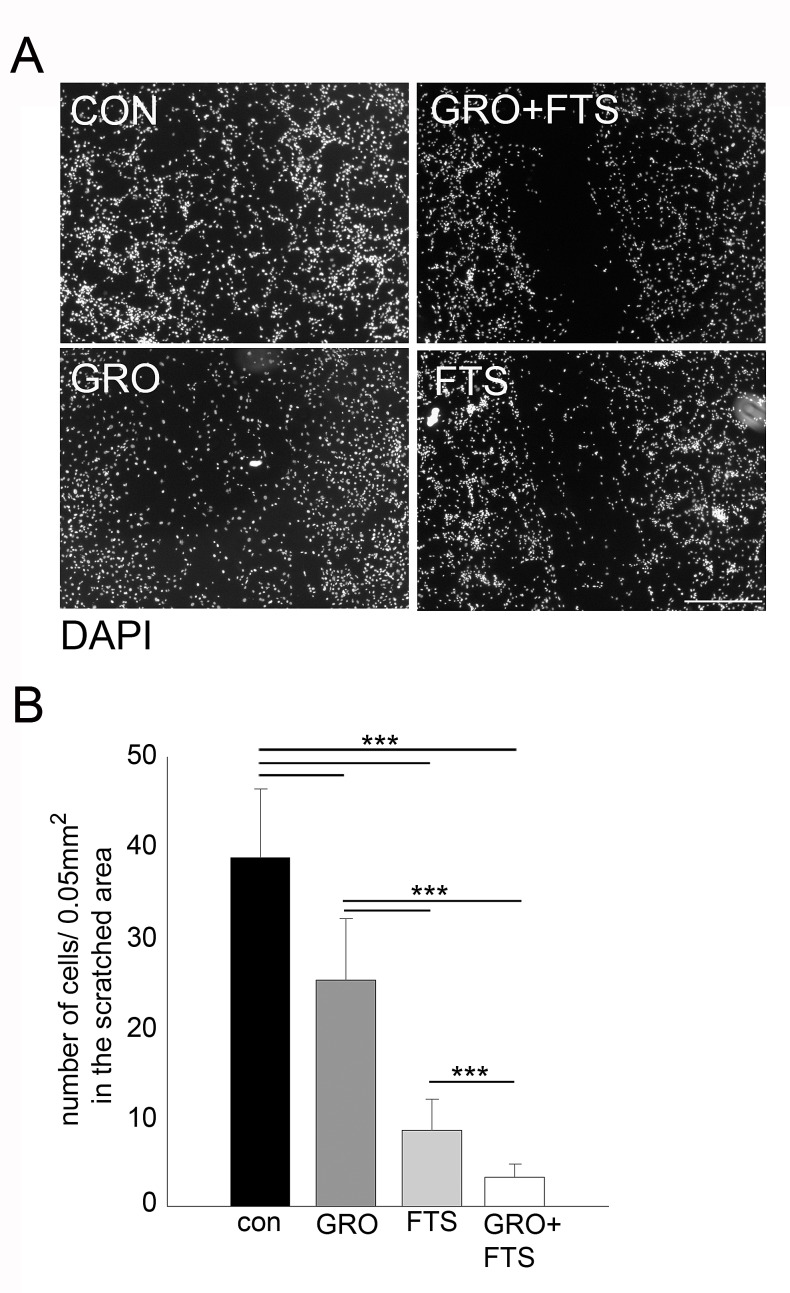
Co-treatment of FTS and GroA affects cell migration U87-MG cells were treated with FTS 75μM, in the presence or in the absence of 10μM GroA. After 48 hr, a scratch wound was inflicted at each well. 24 hr later, the cells were fixed and stained with DAPI. The resulting gap was imaged and cell number in the scratch was quantified and presented as the number of cells in the scratched area (B). Results are mean ± SEM (n =3 experiments; ***p<0.001, one-way ANOVA) in B. Scale bars in A is 200μm.

In order to study the effect of the treatments on cell motility, scratch-induced migration assay was employed [31, 32]. Results of a representative experiment are presented in Figure 5. As shown, both FTS and GroA treatments significantly inhibited the gap closure, with stronger effect induced by FTS treatment. However, the combined treatment exhibited a significantly greater effect compared to each of the treatments alone. These results suggest that, each of the drugs inhibit cell migration, and with better effectiveness when combined.

### FTS and GroA effect on tumor growth *in vivo*

**Figure 6:**
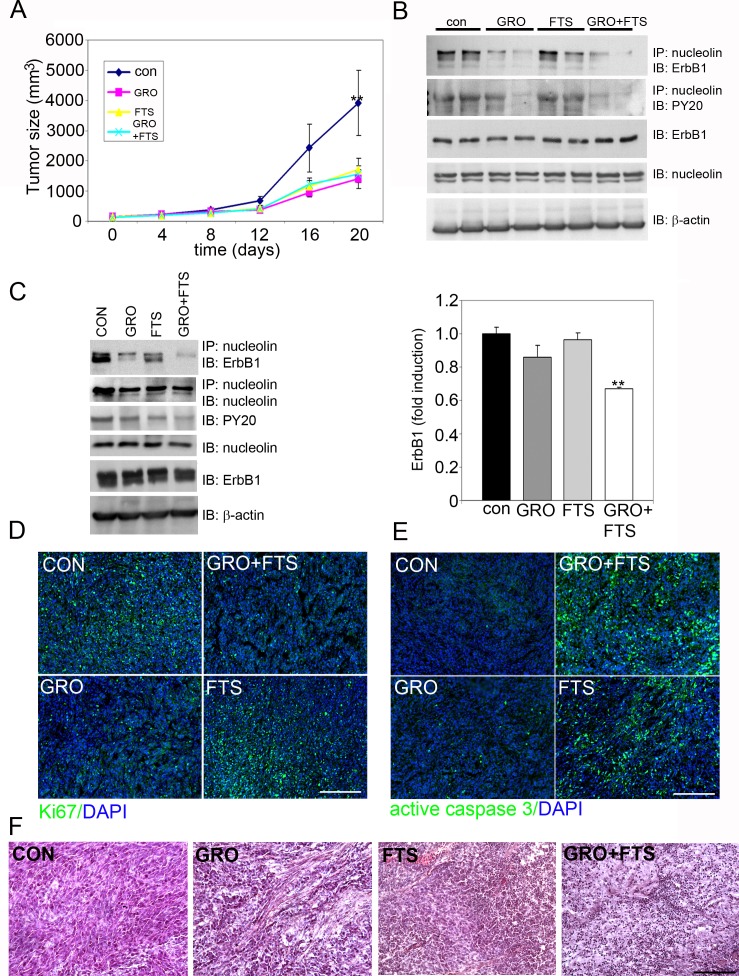
Treatment of FTS and GroA in U87-MG cells *in vivo* significantly inhibited tumor growth The U87-MG cells 2.5 × 10^6^ cells were implanted subcutaneously just above the right femoral joint of nude CD_1_-Nu mice. Tumor volumes were then monitored following the various treatments as described in the M&M. A. Tumor size after 20 days of treatment was significantly reduced in all the treatments compared to the control (n =8 mice; **p<0.01, one-way ANOVA). B. After 12 days of drugs treatment, lysates from tumors were immunoprecipitated with anti nucleolin Ab and blotted with anti ErbB1 or anti PY20. Total tumor lysates were immunobloted with ErbB1, anti nucleolin and with anti-actin Abs. C. After 20 days of drugs treatment, tumor lysates were immunoprecipitated with anti nucleolin Ab and blotted with anti ErbB1 and anti nucleolin Abs. Total tumor lysates were immunobloted with anti-PY20, anti ErbB1, anti nucleolin and with anti-actin Abs. Quantitation of ErbB1 bound to nucleolin is presented, (n =6 mice; **p<0.01, one-way ANOVA). D. Tumors sections prepared 12 days after drug treatment were immunostained with Ki67 (green), and with active caspase 3 (green) (E). F. Tumors sections prepared from mice sacrificed 20 days following treatment were stained with hematoxylin/eosin (H&E). Scale bars in D, E and F is 200μm.

Since FTS and GroA treatments affected cell proliferation and viability, we next wanted to examine whether the treatment has similar effects on tumor growth in nude mice that were xenografted with U87-MG cells *in vivo*. The four groups of mice (8 per group) received 2.5 x 10^6^ cells subcutaneously in the flank. Tumor volumes were monitored every two days. When the tumor reached the volume of approximately 140mm^3^, the mice were randomly divided into four groups; each group was treated with either Cro (oligomer control) + CMC (FTS vehicle), FTS+Cro, GroA+CMC, or FTS+GroA (Figure 6). Twenty days after treatments, the tumors in the groups treated with GroA, FTS and both were significantly smaller then those observed in the control (Cro+CMC) treated mice (Figure 6A). In agreement with the *in vitro* studies, lysates prepared from the tumors showed that the combined treatment reduced nucleolin/ErbB1 interaction as well as ErbB1 phosphorylation at 12 days and at 20 days following treatment (Fig 6 B and C respectively). These results support the conclusion that Ras and nucleolin synergize in receptor activation, which may be related to induction of cell transformation. Furthermore, cell proliferation was inhibited and cell death was enhanced in the combined treatment as evident by immunostaining of the tumor sections with Ki67 and caspase 3 antibodies (Figure 6 D and 6E, respectively). Analysis of tissue morphology depicted by Hematoxylin and Eosin (H & E) staining of the tumor sections revealed that, as opposed to the control sections where cells looked dense and viable, the GroA sections showed reduced cell density with empty spaces. Furthermore, FTS sections showed apoptotic nuclei, while the combined treatment enhanced the effect of each of the drugs alone, exhibiting condensed nuclei and no defined cell structure (Figure 6F). Of note, although there is no significant difference between the treatments in tumor size there are apparent differences in the tissue morphology, cell viability and cell density. Taken together, these results demonstrate drugs synergism on U87-MG xenograft growth inhibition, which are in agreement with the *in vitro* results showing that GroA inhibits proliferation and FTS induces apoptosis.

## DISCUSSION

Glioblastoma are among the most lethal and untreatable types of human adult cancer. Glioblastoma have several genetic and signaling abnormalities that lead to uncontrolled growth, invasiveness, angiogenesis, and eventually facilitate cell proliferation and survival [33, 34]. The impairment of signaling pathways provided the basis for designing molecular-targeted therapy for treatment of glioblastoma. The epidermal growth factor receptor (EGFR)/ErbB1 gene amplification is one of the most frequent alterations, occurring in 30–40% of malignant glioblastoma and it has been associated with tumor invasiveness, angiogenesis, poor survival, and resistance to radiation therapy [35]. ErbB1 activates signaling cascades leading to cell proliferation and it is considered as an attractive target for cancer therapy [36]. Active Ras–dependent pathways are also abundant in most glioblastoma multiform, [37] and contribute to the malignant phenotype by disrupting cell cycle arrest, increasing cell migration, enhancing cell survival, and promoting angiogenesis [38, 39]. The increased presence of active Ras-GTP in glioblastoma is secondary to mitogenic signals originating from activated receptor tyrosine kinases [40]. Nucleolin is highly expressed in glioblastoma and it was shown previously that knocking down nucleolin in glioblastoma cells could inhibit tumor growth and induce cell arrest [15].

In our earlier studies, we have identified a functional crosstalk between Ras, nucleolin and ErbB1 [22, 24]. In the present study we examined the effect of two drugs, directed towards Ras and nucleolin proteins, as a tool to disrupt the synergism between these three oncogenes as a potential treatment for glioblastoma. To target nucleolin we used GroA, and to target Ras, we used FTS. We found a pivotal role of nucleolin in enhancing ErbB1 stabilization, activation and therefore cell proliferation in glioblastoma. Moreover, the combined treatment that targeted nucleolin and Ras (GroA and FTS, respectively) reduced cell growth and viability, more effectively than treatment with each drug alone. GroA mainly inhibited cell proliferation, while FTS induced cell death. Cell motility was inhibited mainly by FTS with smaller contribution of GroA treatment. These results suggest that both drugs can affect different cellular pathways, as they induce different biological responses. Thus a combined treatment may be more efficient in inhibition of glioblastoma cell growth.

Previously, it was demonstrated that FTS attenuates glioblastoma cells by inhibiting both their migration and their anchorage-independent proliferation. The main effect of FTS treatment was mediated by inhibition of phosphatidylinositol 3-kinase signaling [32]. It was also demonstrated that knocking down nucleolin expression inhibits glioblastoma tumor growth [41]. In agreement with these findings, our study suggests a benefit of the combined treatment both *in vitro* and *in vivo.* Each treatment affects slightly different biological responses; FTS mainly affects cell migration and cell viability whereas GroA mainly affects cell proliferation. Moreover, the combined treatment inhibits cell viability, migration and cell proliferation significantly more efficiently than each treatment alone. In addition, the combined treatment inhibits Erk and PKB signaling pathways more effectively than each of the treatments alone. Furthermore, in the *in vivo* experiments, although in the combined treatment the sizes of the tumors were similar to those of the single drug treatments, we found that there was substantial altered tumor morphology. In the combined treatment, the tissue density was reduced with many dead cell regions. This may strengthen the possibility that the combined treatment has a better potential to inhibit tumor cell growth and viability. Taken together, these results are extremely important in light of the absence of drug treatment in brain tumors that express high ErbB1 levels and nucleolin.

In conclusion, our results indicate that treatment with a combination of drugs that target the cooperation between these oncogenes better inhibits tumor cell growth. The combination of FTS and GroA reduces receptor levels and activation, thus inhibiting signaling downstream to the receptor affecting tumorigenicity. These results highlight the superior effect of the combined treatment. Furthermore, our study suggests a new and possibly more effective way to treat cancer patients that overexpressed these oncogenes.

## MATERIALS AND METHODS

### Materials and buffers

FTS (Salirasib, S-*trans, trans*-farnesylthiosalicylic acid) was purchased from Concordia Pharmaceuticals. For FTS preparation, FTS powder was washed in chloroform and the solution was then vaporized by liquid nitrogen twice. The resulted powder was dissolved in 0.1% DMSO in medium supplemented with 10% FBS to concentration of 100mM. The aptamer Gro (GroA/AS1411) and the inactive oligomer Cro, were purchased from IDT (Jerusalem, Israel) as unmodified desalted oligonucleotides. The oligonucleotides were reconstituted in DDW to 1mM concentration and incubated at 65°C for 15 minutes. Methylene blue (1% in boric acid) was purchased from Sigma. HNTG buffer (20mM HEPES pH=7.5, 150mM NaCl, 0.1% Triton X-100, 10% glycerol). Primary antibodies were obtained from the following sources: monoclonal mouse anti-nucleolin C23 (Santa Cruz, sc-8031), polyclonal rabbit anti- ErbB1 antibody (Santa Cruz, sc -03), monoclonal mouse anti-phospho-tyrosine PY20 (Santa Cruz Biotechnology, sc-508), monoclonal mouse anti-actin (MP Biomedicals, 691001), monoclonal mouse anti-BrdU (Roche, 11170376001), polyclonal rabbit anti-pMAPK (cell signaling, 9101S), polyclonal rabbit anti MAPK (cell signaling, 4695S), rabbit anti-pPKB (cell signaling, 4058S), polyclonal rabbit anti PKB (cell signaling, 9272), polyclonal rabbit anti-Ki67 (Thermo Scientific, RM-9106), polyclonal rabbit anti-active caspase 3 (cell signaling, 9664), nuclei were stained with 4,6-diamidino-2-phenylindole (DAPI) (sigma). For BrdU staining, cells were pretreated for 15 min in 2 M HCl before blocking. Secondary antibodies used were: goat anti-rabbit Alexa Fluor 488 (Invitrogen) and goat anti-mouse Alexa Fluor 594 (Invitrogen), polyclonal goat α-Rabbit-HRP (Jackson immunoreaserch, 111-035-144), polyclonal donkey α-mouse-HRP (Jackson immunoreaserch, 715-035-151). For actin staining, cells were stained with 1μg/ml phalloidin-rhodamine (red fluorescence).

### Cell cultures

The human glioblastoma cancer cells, U87-MG, were grown in Dulbecco's modified Eagle's DMEM (Biological Industries) supplemented with antibiotics, 1% L-Glutamine and 10% heat-inactivated fetal bovine serum (FBS; Hyclone, Thermo Scientific). The cells were incubated at 37°C in 5% CO_2_ in air, and the medium was changed every 3-4 days. One day before treatment the cells were plated at ~50% confluence in medium supplemented with 5% fetal bovine serum. Treatments with FTS, with or without GroA, were according to the indicated concentration (cells were treated with 0.1% DMSO as control for FTS and Cro as a control for GroA) for the times specified in each experiment.

### Lysates preparation, immunoblot and immunoprecipitation analysis

U87-MG cells were plated into 10cm culture dish at 1×10^6^ cells in phenol DMEM supplemented with 5% FCS, 24hr before treatment. After treatment, cells were lysed and protein concentration was determined using Bradford assay (BioRad). Equal amount of protein was taken for each immunoprecipitation or immunoblot. For immunoprecipitation, monoclonal antibodies were first coupled to anti-mouse IgG agarose (Sigma), for 30 min at 4°C, then the proteins in the lysate supernatant were immunoprecipitated with aliquots of the beads-antibody complexes for 2 h at 4°C. The immunoprecipitates were washed with HNTG buffer, resolved by SDS-polyacrylamide gel electrophoresis (PAGE) through 7.5% gels and electrophoretically transferred to nitrocellulose membrane. For immunoblot analysis, equal amounts of protein from each sample were loaded and resolved by SDS-polyacrylamide gel electrophoresis through 7.5%-10% gels. The gels were electrophoretically transferred to a nitrocellulose membrane. Membranes were blocked, blotted with the corresponding primary antibodies followed by secondary antibody linked to horseradish peroxidase. Immunoreactive bands were detected by chemiluminescence reaction. The protein levels were quantified by a densitometric analysis of protein bands using the ImageJ software.

### Assays of cell survival and cell death

U87-MG cells were plated in medium supplemented with 5% FBS, treated with or without FTS, GroA or Cro for the indicated time. Cell numbers were determined by the methylene blue assay. For this purpose, the cells were fixed with 4% formaldehyde in phosphate-buffered saline for 2 hours, then washed once with 0.1 M boric acid (pH 8.5) and incubated with the DNA-binding dye methylene blue (1% in boric acid) for 20 minutes at room temperature. The cells were then washed and lysed with 0.1 M HCl. Absorbance was measured with a Tecan Spectrafluor Plus spectrophotometer (Mannedorf, Switzerland) at 595 nm. Cell viability was calculated as the ratio of absorbance in treated cultures to that in untreated control cultures. Nuclear staining and nuclear morphology scored dead cells. To estimate the number of dying cells, live cells were incubated for 5 minutes with 1μg/ml of the fluorescent DNA dye bisbenzimide (Hoechst 33258; Sigma). As a positive control, the cells were treated with 200 nM STS (staurosphorine). After staining, the cells were photographed with an Olympus motorized inverted research microscope Model IX81 (20× magnification). In each field (10–15 fields for each treatment) the percentage of dying cells was estimated by counting the Hoechst-positive cells relative to the total number of cells (100–200 cells per field), and expressing the result as a percentage of the total cell number.

### Immunohistochemistry

U87-MG cells were seeded on coverslips coated with poly-L-Lysine in DMEM supplemented with 5% FBS. The cells were then stimulated with the indicated treatments and time and fixed with 4% PFA (paraformaldehyde) for 10 min followed by incubation in blocking solution (PBS-Triton X-100 containing 5% normal goat serum; Jackson immunoreaserch) for 1 hr at room temperature. Primary antibodies were diluted in blocking solution and cells were incubated for 1 hr in room temperature. After washing, cells were incubated for 1 hr at room temperature with secondary antibodies diluted in blocking solution. Cells were mounted in Fluoromount (Dako).

### Microscopy

Cells were examined under a fluorescence microscope at 10× to 60×magnification (as indicated) with Olympus motorized inverted research microscope Model IX81. Average fluorescent intensity after immunostaining against p-MAPK and p-PKB were quantified using ImageJ.

### Scratch-induced migration assay

Cells were plated in six-well plates. Two days after treatment, by the time confluency was reached, a scratch wound was inflicted at each well. 24 hour later, the cells were fixed with 4% PFA in PBS, followed by nuclei labeled with DAPI. The resulting gap was imaged and cell number in the scratch was quantified.

### BrdU-positive cell counts

Cells were examined under a fluorescence microscope at 20×magnification with Olympus motorized inverted research microscope. In each field (15-20 fields for each treatment) the percentage of proliferating cells was estimated by counting the BrdU-positive cells relative to the total number of cells (DAPI labeled). (100–300 cells per field), and expressing the result as a percentage of the BrdU- positive cell number.

### Tumor Growth in Nude Mice

The study was conducted according to the NIH Guidelines for Use and Care of Laboratory Animals and following the approval by Animal Care Committee of the Tel Aviv University. U87-MG cells were implanted into nude nice. Nude CD_1_-Nu mice (25-30 g) were housed in barrier facilities on a 12-h light/dark cycle.

On day zero, 2.5 ×10^6^ cells in 0.1 ml of PBS were implanted subcutaneously just above the right femoral joint. Tumor size was measured every 4 days. When the tumors were apparently seen and the calculated tumor size was 140 mm^3^, the animals were divided randomly into four groups of mice (control, FTS treatment, GroA treatment and combined treatment; 8 mice per group). GroA treatment was performed by intraperitoneal injection of 4.5 mg/kg in 100 μl PBS (or 4.5 mg/kg Cro in 100 μl PBS for control mice) every other day. FTS was orally administered daily at 60 mg/kg, and was prepared in H_2_O/0.5% carboxymethyl cellulose (CMC; or H_2_O/0.5% CMC for control mice). Tumor volumes were monitored on the indicated days as described previously [42]. At the end of the experiment, the mice were sacrificed and the tumors were dissected and used for protein analysis and immunohistochemistry.

### Tumor analysis

Every tumor was cut in half with a scalpel blade; half was taken for protein analysis and half for immunohistochemistry and histology. For protein analysis, tumors were homogenized by a polytron homogenizer in lysis buffer, immunoblot and immunopercipitations were done as described above. For immunostaining tumors were post-fixed for 3 hours in cold 4% PFA followed by 20% sucrose in PBS overnight at 4^0^C. Cryostat sections (20 μm) were cut and stained using standard immunohistochemistry as described above. Primary antibodies: rabbit anti-Ki67 (1:300, Thermo Scientific), rabbit anti-active caspase 3 (1:1000, R&D Systems). Standard protocol was used for H&E staining.

### Statistical analysis

All experiments were performed at least 3 times unless otherwise stated. Experimental differences were tested for statistical significance using one-way ANOVA followed by the Tukey test for multiple comparisons with α=0.001, and Student's t-test with 95% confidence when comparing two parameters. P-value of <0.05 was considered as significant.
